# Removal of Fecal Indicators, Pathogenic Bacteria, Adenovirus, *Cryptosporidium* and *Giardia* (oo)cysts in Waste Stabilization Ponds in Northern and Eastern Australia

**DOI:** 10.3390/ijerph13010096

**Published:** 2016-01-02

**Authors:** Maxim Sheludchenko, Anna Padovan, Mohammad Katouli, Helen Stratton

**Affiliations:** 1 Smart Water Research Centre, Building G51, Griffith University, Southport, Queensland 4222, Australia; h.stratton@griffith.edu.au; 2 Genecology Research Centre, University of the Sunshine Coast, Sippy Downs, Queensland 4558, Australia; MKatouli@usc.edu.au; 3 Research Institute for the Environment and Livelihoods, Charles Darwin University, Darwin, Northern Territory 0909, Australia; Anna.Padovan@cdu.edu.au

**Keywords:** waste stabilization pond (WSP), maturation pond, *E. coli*, enterococci, *Campylobacter jejuni*, *Salmonella enterica*, *Giardia* cysts

## Abstract

Maturation ponds are used in rural and regional areas in Australia to remove the microbial loads of sewage wastewater, however, they have not been studied intensively until present. Using a combination of culture-based methods and quantitative real-time PCR, we assessed microbial removal rates in maturation ponds at four waste stabilization ponds (WSP) with (*n* = 1) and without (*n* = 3) baffles in rural and remote communities in Australia. Concentrations of total coliforms, *E. coli*, enterococci, *Campylobacter* spp., *Salmonella* spp., F+ RNA coliphage, adenovirus, *Cryptosporidium* spp. and *Giardia* (oo) cysts in maturation ponds were measured at the inlet and outlet. Only the baffled pond demonstrated a significant removal of most of the pathogens tested and therefore was subjected to further study by analyzing *E. coli* and enterococci concentrations at six points along the baffles over five sampling rounds. Using culture-based methods, we found a decrease in the number of *E. coli* and enterococci from the initial values of 100,000 CFU per 100 mL in the inlet samples to approximately 1000 CFU per 100 mL in the outlet samples for both bacterial groups. *Giardia* cysts removal was relatively higher than fecal indicators reduction possibly due to sedimentation.

## 1. Introduction

Wastewater stabilization pond (WSP) systems are a cost efficient option for urban domestic wastewater treatment, and are widely utilized globally for the treatment of domestic sewage and grey waters [1]. Since the operation of WSPs does not involve the use of expensive equipment for aeration, pumping, building of concrete structures or other mechanical processes, these systems are also an attractive option for use in rural and remote communities.

A typical WSP system consists of three consecutively connected reservoirs: anaerobic or Imhoff tank, facultative and maturation (stabilization or polishing) ponds. Maturation ponds are the last stage of WSP systems and are typically about one meter in depth and capable of almost 3 log pathogen removal efficiency [2] but have not been intensively studied in Australia. In real practice, to increase the efficiency of maturation ponds, there are a number of other pre- and post-treatment steps which includes Imhoff tank, constructed wetlands and rock filters [2]. To comply with State and Territory faecal load regulations, additional treatment after the maturation pond is usually required to bring microbial indicator concentrations down to acceptable levels. For instance in Australia, constructed wetlands (CW), reed beds (RB) or microfiltration plants are commonly used to augment WSP systems [3]. However, regulators often allow effluent from maturation ponds to be released directly into the environment, which potentially causes a health risk to local communities.

Each WSP system is different in design, influent composition, pre-treatment type and located in different climates, so pathogen removal rates can vary from site to site. Currently, total coliforms, faecal coliforms and more recently, *E. coli* concentrations, are monitored in effluent wastewater to indicate pathogen removal [4]. However, faecal indicators do not always reflect concentrations of pathogens, particularly viruses, in effluent [5,6]. Many WSPs systems are located in remote areas where microbial monitoring of water quality is hard to perform. Due to lack of simple laboratory facilities and a long time required for samples delivery to the closest laboratory, the assessment of microbial treatment efficiency of such WSPs is difficult to be done. Therefore, to validate the quality of effluent over time, multiple datasets are required due to seasonal variation which are normally associated with high costs of sampling and analysis. Furthermore, delivery of samples to be processed at accredited water quality centers remains the main limitation for pathogen validation in such systems. Under these conditions, storage of samples for long periods of time is the only option for later analysis. The application of modern molecular methods would help to improve the modelling, designing and maintaining remote WSP systems, as more data on pathogens can be provided from multiple sampling events at relatively low cost. Quantitative PCR allows the analysis of many target pathogens from long-term stored frozen samples. To our knowledge, such an approach has not been applied for the assessment of bacterial and to some extent viral quality in WSP systems and is certainly not carried out routinely.

In the present study, we evaluated concentrations of several common faecal indicators as well as key pathogens using a combination of culture–based and q-PCR methods over a period of two years from four WSPs in Australia. Australian water recycling guidelines (AWRG) recommend that indicator organisms (e.g., faecal coliforms, *E. coli*, or enterococci) and reference pathogens (*Campylobacter* spp., adenovirus, and *Cryptosporidium* and *Giardia*) data should be collected for assessment of microbial risks related to recycled water [7]. The aims of this study were: (a) to compare the performance of WSP systems from different locations with a focus on maturation ponds as a main component for pathogen removal; (b) to compare culture *vs.* molecular methods in assessing indicator; (c) to compare pathogen concentrations and log removals; and (d) to follow the dynamics of pathogen removal within a maturation pond and subsequent post-treatment in constructed wetlands and reed beds.

## 2. Experimental Section

### 2.1. Sampling Sites

**Figure 1:**
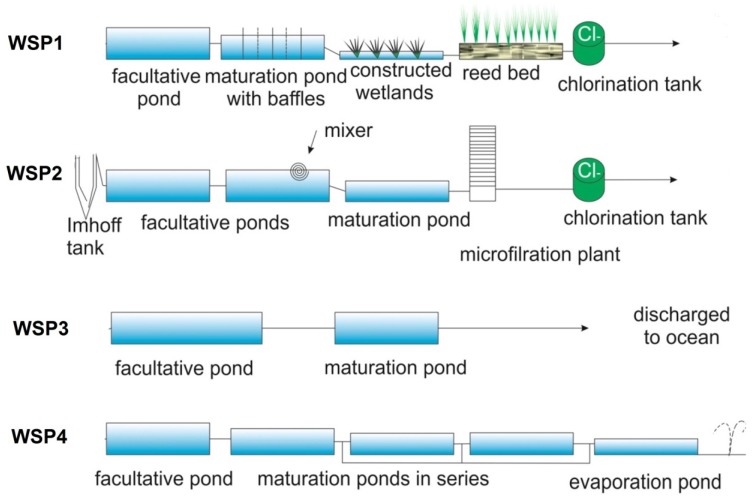
Schematic representation of maturations ponds studied within four different WSP systems. Maturation ponds of WSP1 and WSP2 were located in rural area of South East Queensland and had post-treatment such as constructed wetlands, reed beds and microfiltration plant with further chlorination of final effluent. WSP3 and WSP4 were located in remote aboriginal communities of the Northern Territory. Treated effluent from WSP3 maturation pond was discharged directly via an ocean outfall. WSP4 had 3 maturation ponds, with only the first studied here; effluent was the polished in an evaporation pond with further irrigation (sprinkling) on adjacent land.

Four WSP systems were investigated. These include two in subtropical South East Queensland—WSP1 and WSP2, and two in wet-dry tropic regions of the Northern Territory. WSPs 1 and 2 are located in a sub-urban community with a population around 1500 inhabitants, WSPs 3 and 4 are located in remote aboriginal communities serving 1000–2500 inhabitants. WSP1 (Figure 1) consists of a primary 65 × 65 × 1.5 m facultative pond, a 30 × 60 × 1.2 m baffled maturation pond with 12–20 days retention time, two 30 × 60 m constructed wetlands (CW) and three 15 × 20 m reed beds (RB). Sampling points H1 to H6 indicate sampling sites in the baffled maturation pond. The effluent from the outlet of the maturation pond enters the CWs which are covered with the macrophytes *Baumea rubiginosa, B. articulate*, *Bolboschoenus caldwelli*, *Eleocharises phacelata* and *Schoenoplectus validus*. The effluent from CWs flows into the dry RBs that are covered with *Phragmites australis*.

WSP2 consists of three ponds without baffles (see Figure 1). The raw sewage enters into Imhoff tank which flows into pond 1 (facultative pond). From this pond, the effluent enters pond 2 which is equipped with an aeration pump. A third pond (a maturation pond; 60 × 32 ×1 m, ~10 h of retention time) from which samples were collected, receives wastewater from pond 2. Finally the wastewater from pond 3 enters a micro-filtration plant and after passing through chlorination tank, the water enters a reservoir lagoon (see Figure 1).

WSP3 consists of a primary treatment (facultative) pond which receives raw sewage and a maturation pond (65 × 40 × 1.2–2 m, estimated <1 h of retention time). The treated wastewater is discharged via an ocean outfall. Finally, WSP4 consists of a primary treatment pond (facultative), three consecutive maturation ponds of similar size (60 × 30 × 1.2 m, with an estimated 3.3 days of retention time each), and a final evaporation pond. The effluent is sprayed onto the surrounding land by sprinkler irrigation (see Figure 1).

*Sampling from WSP1.* The first round of sampling was carried out on 3 October 2013. Grab samples were collected in 500 mL bottles for bacterial and viral analysis and 1 L bottles for protozoa directly from the inlet pipeline of the influent (*i.e*., Inlet), at 30 cm depth of the effluent pond at two and a half meters from the inlet pipeline (H1), the edge of each baffle (H2 to H6) as well as the outlet pipeline (*i.e*., Outlet) (see Figure 1). An additional four samples were collected from the same sites in March, May, July and September 2014. For 2014 sampling events, the outlet pipe had been shifted making it difficult to reach for sampling and therefore effluent samples were collected within two meters from the outlet.

*Sampling from WSP2.* Sampling was done on 11 September 2013 and on 16 July 2014. The inlet pipeline is situated 20 cm below the surface of the water and two meters inside the pond. Grab samples were collected as close as possible to the inlet pipeline. Effluent samples were collected before the microfiltration plant facility.

*Sampling from WSP3.* Grab samples were collected on 11 October 2013 and 4 September 2014 near the inlet and outlet sites of the maturation pond.

*Sampling from WSP4*. Grab samples were collected on 3 June 2014 from the cemented wells located before the inlet and after outlet locations of the first of three maturation ponds.

### 2.2. Physico-Chemical Measurements

Physico-chemical parameters (temperature, dissolved oxygen (DO), pH, conductivity and turbidity) were measured using a Hydrolab DS-5 YSI probe (Hach Environmental, Loveland, CO, USA) (Table 1). Stratification was observed in WSP3 (results not shown).

**Table 1:** Physico-chemical characteristics of sample sites during samplings.

Ponds	Zone	Year	T °C	pH	DO, mg/L	Turbidity, NTU	Conductivity, µS/cm
WSP1	subtropical	2014	17–22	8.8–9.4	8–23.7	26–72	842–1041
WSP2	subtropical	2014	13.6–17.5	10.2–10.6	17.4–24.4	34–66	929–1002
WSP3	tropical	2013	28.7–38.0	6.08–10.45	0–34.26	76–136	467–766
WSP4	tropical	2014	25–26.4	7.8–9.2	9.9–11.4	27–65	1750–1876

Samples for bacterial and phage analyses were collected in sterile 500 mL polypropylene bottles in duplicate. Samples were stored on ice and analysed within eight hours of collection. Samples for the detection of protozoa and adenovirus were collected in two 5 L polypropylene containers.

### 2.3. Culture Based Bacterial Quantification

Total coliforms, *Escherichia coli*, enterococci, *Salmonella* spp. and *Campylobacter* spp. were enumerated using the membrane filtration technique [8] with some modifications. Individual wastewater samples were mixed thoroughly, serially diluted in PBS or sterile distilled water (treatment provided no difference in counts) and filtered through 0.45 µm sterile mixed cellulose ester membrane filters (Advantec, Tokyo, Japan) using a CombiSart^®^ manifold filtration unit (Sartorius, Gottingen, Germany). For enumeration of *E. coli*, the filter was placed on modified mTEC agar plates (BD, Sparks, AR, USA) incubated at 35 °C for 2 h followed by an additional incubation at 44.5 °C for 24 h. Single magenta colonies were quantified and reported as *E. coli* numbers. For enumeration of enterococci, filters were placed on mEI agar plates (BD) supplemented with 0.024% (*w*/*v*) of nalidixic acid (Sigma-Aldrich, St Louis, MO, USA) and plates were incubated at 41.5 °C for 24 h. Colonies with blue halos were regarded as enterococci. Enumeration of total coliforms was done by placing filters onto Chromocult^®^ coliform agar plates (Merck, Darmstadt, Germany) and plates were incubated at 35 °C for 24 h. The sum of salmon to red colonies and dark blue colonies were reported as total coliforms. *Salmonella* spp. were enumerated by two approaches. The first method included membrane filtration, with filters placed on XLD agar plates (Oxoid, Hampshire, UK) and incubated at 35 °C for 48 h. Colonies which had the same color as the media, translucent and with or without black centers were reported as *Salmonella* spp. The second method was the mini MPN technique [9] using brilliant Rappoport-Vasiliadis liquid media (Oxoid). Briefly, 30 mL of wastewater sample was enriched for 24 h at 37 °C with peptone water (Oxoid) in a 50 mL plastic tube. One mL of the enrichment was added into a 12 well plate containing brilliant liquid selective media and incubated with shaking for 4 h at room temperature. 50 µL from each incubated well were then serially transferred to a fresh 12 well plate with the same selective media and plates were incubated at 37 °C overnight. Visually yellow colored and having high turbidity wells were assumed to be positive for *Salmonella* spp. For enumeration of *Campylobacter*, filters were placed on *Campylobacter* mCCDA agar (Oxoid) with selective supplement SR0155 (Oxoid). Plates were incubated at 43 °C for 48 h under microaerofilic conditions using the Campygen system (Oxoid). All colonies were counted as thermophilic *Campylobacters* which include human pathogens *C. jejuni* and *C. coli*.

*E. coli* ATCC 15766, *Enterococcus faecalis* ATCC 19433, *Salmonella enteric* serovar Typhimurium, *Campylobacter jejuni* NCTC 11168 were used as positive controls to confirm the performance of selective medium. All medium supported growth of specific bacteria providing relevant morphological characteristics.

### 2.4. F+ RNA Coliphage Quantification

Male-specific (F+) RNA coliphages were enumerated according to the APHA 9224B double-agar-layer method [8]. Briefly, an overnight host strain *E. coli* ATCC 700891 was subcultured into fresh tryptic soy broth, grown for 4 h at 37 °C and kept on ice to avoid losing pili. Assay tubes contained 3 mL of soft 0.7% tryptone agar (BD) with 1.5 mg/mL each ampicillin and streptomycin and 100 µL of host strain, were kept at 47 °C in a waterbath. To this mixture, 1 mL of wastewater sample was added. The contents of the tube were mixed gently and poured onto plates with 1% (*w*/*v*) tryptone agar supplemented with antibiotics. After the top agar layer was solidified, plates were incubated at 36 °C overnight. Plaque counts were reported as PFU/100 mL. A diluted stock of 1 × 10^2^ PFU/100 mL of MS2 phage ATCC 15597-B1 was used as positive control. Tryptone broth with positive control phage provided plaques with *E. coli* ATCC 700981, while tryptone broth without phage allowed forming lawn of host strain on agar plate. 

### 2.5. Viral Precipitation and Infectivity Assay

Viral particles in 1L wastewater samples were precipitated in 8% PEG 6000 with 1% Tween^®^ 20 (Sigma-Aldrich) and 0.5% 1 M·CaCl_2_. After an overnight incubation at 4 °C, samples were centrifuged at 10,000 g for 30 min at 4 °C. Pellets were washed with 5 mL of PBS and eluted for an hour with periodic vortexing. The final 10 mL was extracted with equal volume of chloroform and stored at −80 °C. Two hundred microliters of the eluted sample was used for DNA extraction using the DNeasy Blood and Tissue Kit (Qiagen, Dusseldorf, Germany). Most Probable Number of Infectious Units (MPNIU) was determined according to the cell culture assay standard procedure [10] with modification by in-house method MP568 in accredited laboratory (ALS, Scoresby, Australia). Briefly, infectivity was assessed by propagation of A549 human cell line with serial dilutions of viral PEG concentrates in a 10-mL cell culture flask MPN format. The detection limit for the infectivity assay was 1.1 MPNIU.

### 2.6. Cryptosporidium and Giardia Enumeration

ColorSeed (BTF, Sydney, Australia) stained with TexasRed used as an internal control was spiked into 1 L of wastewater sample prior to filtration. Samples were filtered through Filta-Max membrane units (IDEXX, Westbrook, ME, USA) according to the manufacturer’s guidelines. Routine elution using the Filta-Max manual wash station (IDEXX) and immune magnetic separation (IMS) concentration steps were conducted according to the manufacturer’s protocols using Dynabeads^®^GC Combo Kit (IDEXX) containing monoclonal antibodies specifically binding to oocysts. The final 50 µL of IMS concentrate was fixed onto a microscope slide, stained with DAPI (4′,6-diamidino-2-phenylindole dihydrochloride) and EasyStain (BTF) containing FITC-labelled antibodies and stained (oo)cysts visualised by epifluorescent microscopy (Nikon, Tokyo, Japan). (Oo)cyst numbers were estimated based on recoveries of ColorSeed. According U.S. EPA 1623 method, recovery with more 11% is accepted as sufficient to report numbers of (oo)cysts.

### 2.7. DNA Extraction and Recovery

Between 10 and 100 mL of wastewater were filtered onto 47 mm 0.45 µm mixed cellulose ester filters (Advantec, Tokyo, Japan) and stored at −20 °C. Filters were aseptically cut and placed into 2 mL microcentrifuge tubes for nucleic acid extraction steps following the instructions given by each manufacturer (see below). Several nucleic acids extraction kits were applied including PowerWater DNAand PowerSoil RNA/DNA (MoBio, Carlsbad, CA, USA), FastDNA SPIN kit for Soil (MP Biomedicals, Santa Ana, CA, USA), MasterPure Complete DNA and RNA (Epicentre, Madison, WI, USA), DNeasy Blood and Tissue Kit and QIAamp DNA stool mini kit (Qiagen).The FastDNA SPIN kit for Soil demonstrated the best recoveries values based on DNA yield for enterococci and *E. coli* (data not shown) and was used to isolate genomic DNA from all wastewater samples. An initial cell disruption step for this kit was conducted using a Mini-Beadbeater-16 (BioSpec, Bartlesville, OK, USA) for 60 s.

### 2.8. Preparation of Stocks for qPCR Calibration Curves

*E. coli* ATCC 15766*, Enterococcus faecalis* ATCC 19433 and *Salmonella enterica* serovar Typhimurium (isolated and identified in our laboratory) were enriched overnight in Brain Heart Infusion (Oxoid) broth. *C. jejuni* NCTC 11,168 was enriched in Preston media containing defibrinated horse blood in Hungate tubes under microaerophilic conditions. Cells were harvested by centrifugation at 3000 g for 10 min, washed with 1× PBS and DNA was extracted with the FastDNA SPIN kit for soil (MP Biomedicals).

Plasmid vector for adenovirus detection was constructed and used to quantify all serotypes of adenovirus. Briefly, adenoviral DNA was kindly provided by Dr. Jatinda Sidhu (CSIRO, Brisbane, Australia) and amplified with hexon gene primers (Table 2). The resulting 130 bp PCR product was ligated into pGEM^®^ T-Easy Vector (Promega, Madison, WI, USA) according to standard protocols. Clones were grown in LB broth (BD) and the plasmid was purified with QIAprep Spin miniprep kit (Qiagen). Dilutions of purified stock of plasmid pAdV (3145 bp) was used to generate standard curves for adenovirus enumeration.

DNA concentrations were assessed by BioPhotometer plus (Eppendorf, Hamburg, Germany) with 1 mm Hellma TrayCell microcell (Hellma Analytics, Mullheim, Germany) or Nanodrop (Thermo Scientific, Sydney, Australia). Genomic DNA was serially diluted to give a range of 10^7^ to 10^1^ genome copies. To calculate genome copies from known amount of DNA, sizes of reference genomes were obtained from the NCBI database. It was assumed that each base pair of DNA mass is 1.096 × 10^−21^ g. For instance, the mass of a single copy genome of 4,746,218 bp for *E. coli* ATCC 15766 would weigh 5.20185 × 10^−15^ g or 52.0185 × 10^−5^ ng. Hence, an initial stock of 10^8^ gene copies should contain 520.185 ng of DNA extract. Genome sizes of *Enterococcus faecalis*, *Salmonella enterica*, and *Campylobacter jejuni* were assumed to be 3,359,974 bp, 4,964,097 bp and 1,641,481 bp respectively.

### 2.9. PCR Primers and TaqMan Probes

QPCR assays were used to enumerate the following organisms: *E. coli*, *E. faecalis*, *S. enterica*, *C. jejuni* and adenovirus. All primers and probes (see Table 2) were selected from previously published data except for the *gyr*B probe for *S. enteric* enumeration, which was designed using the online PrimerQuest tool available at the IDT web-site [11]. The specificity of this probe was verified by performing a BLAST search of Genbank [12] and by running qPCR with *S. enterica* serovar Typhimurium strain. Primers and probes were purchased from Macrogen (Seoul, Korea) or from IDT (San Diego, CA, USA). A DNA internal amplification control—phagemid pM13mp18 [13], was tested at concentrations ranging from 10^1^ to 10^5^ gene copies together with all standard DNA markers. 

### 2.10. Real-Time qPCR

Each 25 µL of qPCR reaction mixture contained 12.5 µL of 2× GoTaq Probe qPCR Master Mix (Promega), 400 nM forward, 400 nM reverse primers and 200 nM of hybridization probe for target gene, and 400 nM of forward, 400 nM of reverse primer and 200 nM of probe for internal amplification control (IAC), 10^3^ copies of IAC, DNAse-RNAse free water, and 5 µL of DNA template. Real-time PCR reactions were placed in a CFX96 thermal cycler (Bio-Rad, Hercules, CA, USA) with an initial polymerase activation of 95 °C for 10 min, followed by 40 cycles of 95 °C for 10 s, 60 °C for 1 min. Standard DNA markers of 10^5^ to 10^1^ gene copies were run in triplicate reactions along with no template controls. Reaction assays for unknown samples were run in duplicates. For qPCR assays, the PCR efficiency calculated from calibration curves were within 90%–105%. No inhibition was observed for any of the microorganisms tested as determined by no change in the cycle times of the internal control in the presence or absence of DNA extracted from wastewater. LOD for *E. coli* and enterococci qPCR was 10 and 40 gene copies respectively. LOQ was 100 genome copies for both microorganisms.

**Table 2:** Sources and concentrations of primers and probes with fluorescence label used for qPCR assays to quantitatively detect path.

Microorganisms	Gene	Function	Sequences (5′-3′)	Primer Concentration, µM	Reference
*E. coli*	*uidA*	glucuronidase	F: GTGTGATATCTACCCGCTTCGC	0.7	[14]
			R: AGAACGGTTTGTGGTTAATCAGGA	0.7	
			P: TCGGCATCCGGTCAGTGGCAGT	0.2	
*Enterococcus faecalis*	*23s RNA*	Ribosomal gene	F: GAGAAATTCCAAACGAACTTG	0.5	[15]
			R: CAGTGCTCTACCTCCATCATT	0.5	
			P: TGGTTCTCTCCGAAATAGCTTTAGGGCTA	0.08	
DNA IAC pM13mp18	NA		F: AAGATTTGAATCGGTTGCTTGG	0.4	[13]
			R: GCCACTGCTCCATTATCTGG	0.4	
			P: CCGATTGTTAGCCAGCCCATGCCA	0.2	
Adenovirus (all types)		Hexon gene	F: GCCACGGTGGGGTTTCTAAACTT	0.5	[16]
			R: GCCCCAGTGGTCTTACATGCA	0.5	
			P: TGCACCAGACCCGGGCTCAGGAGGTACTCCGA	0.2	
*Campylobacter jejuni*	*mapA*	Mucus adhesion-promoting protein	F: GGTTTTGAAGCAAAGATTAAAGG	0.5	[17]
			R: AAGCAATACCAGTGTCTAAAGTGC	0.5	[17]
			P: TGGCACAACATTGAATTCCAACATCGCTA	0.3	[18]
	VS1	n/a	F: GAATGAAATTTTAGAATGGGG	0.4	[19]
			R: GATATGTATGATTTTATCCTGC	0.4	
			P: TTTAACTTGGCTAAAGGCTAAGGCT	0.1	
*Salmonella enterica*	*gyrB*	gyrase protein	F: CGTGGGCGTCTCGGTAGTY	0.5	[20]
			R: CTCATATTCAAATTCAGTGACG	0.5	[20]
			P: AAACCGGCACGATGGTACGTTTCT	0.25	This study
	*ttrRSBCA*	tetrathionate respiration	F: CTCACCAGGAGATTACAACATGG	0.4	[21]
			R: AGCTCAGACCAAAAGTGACCATC	0.4	
			P: CG +ACG +GCG +AG+ACCG	0.25	

### 2.11. Identification of Campylobacter *spp.*

To confirm the identity of colonies growing on the mCCDA plates, DNA was extracted from six grey colonies from each mCCDA agar plate according to a protocol described previously [22] and 5 µL of template was added in a qPCR assay specific for *C. jejuni* [19]. DNA extracted from *C. jejuni* NCTC 11168 was used as a positive control.

### 2.12. Statistical Analysis

Wilcoxon matched-pairs signed rank test was used to compare differences between influent and effluent microbial concentrations of each pond. The test was conducted in GraphPad Prism v6.02 (Software MacKiev, Kiev, Ukraine). Pearson and Spearman correlation was used to calculate relationship between qPCR and CFU values and tests were undertaken in SPSS Statistics v22 (IBM, Armonk, NY, USA). A *p*-value of <0.05 was considered statistically significant.

## 3. Results

### 3.1. Concentrations of Microorganisms in Maturation Ponds

**Figure 2:**
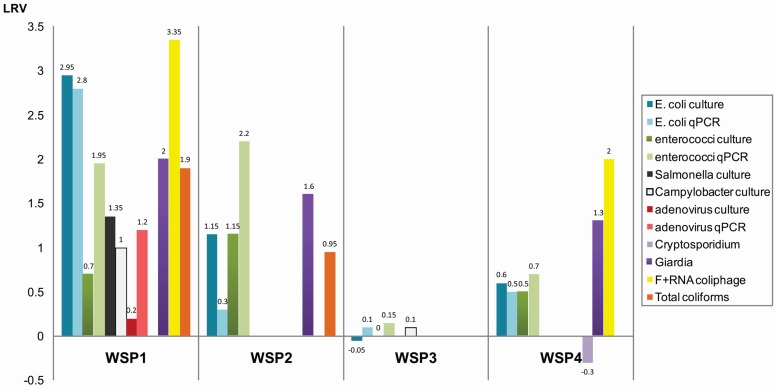
Average removal of microorganisms in maturation ponds located at four WSPs and tested during two years. Graph summarizes log removal values (LRV) presented in Table 3. Data on qPCR for *Salmonella enterica* and *Campylobacter jejuni* are not shown due to lack of detection of these pathogens in the maturation ponds. Removal of *Cryptosporidium parvum* are not shown due to absence of oocysts in effluent of WSP1. Samples taken from WSP2, WSP3 and WSP4 were tested only partially, blanks are for microorganisms which were not tested.

WSP1 demonstrated the best performance based on the significant statistical differences (*p* < 0.0001) found between the number of bacteria, coliphages, adenovirus and pathogenic protozoa in samples from the inlet and the outlet of the pond. In this WSP, the numbers of *E. coli* were as high as 1.5 ± 0.2 × 10^5^ CFU/100 mL in influent samples but decreased to 3.5 ± 0.8 × 10^2^ CFU/100 mL in effluent samples in 2014 and sometimes dropped to 26 ± 0.7 CFU/100 mL in 2013, providing around 3 log reduction (Table 3, Figure 2). High reductions were observed in concentrations of *Giardia* cysts (2 log) and F+ RNA coliphage (3 log) in effluent samples. Less reduction (1.2 log) was measured for adenovirus with initial concentrations in the influent of >2.3 MPNIU per 1 L. A reduction in the numbers of *Salmonella* spp. in this WSP was lower than *E. coli* and varied between 1.2–1.5 log (Table 3). Concentrations of thermotolerant *Campylobacter* spp. in influent (70 CFU/100 mL), while relatively low, dropped even further in effluent samples (6 CFU/100 mL). The number of enterococci did not decrease as much as *E. coli* and *Salmonella* (Table 3).

QPCR showed a log higher number of *E. coli* and enterococci in both influent and effluent samples compared to culture-based methods, but the level of reduction in the number of these bacteria using both methods did not differ (Table 3). Concentrations of adenovirus DNA measured by qPCR gave much higher numbers (4.8 ± 1.5 × 10^5^ genome copies per 100 mL) than culture-based assay in influent samples. Adenovirus concentrations in the effluent were below the level of quantification/detection.

WSP2 is of similar size and in the same climate zone as WSP1 but lacks baffles. The microorganisms tested in WSP2 showed no significant reduction in numbers between inlet and outlet either by culturing or qPCR in 2013. However, due to occasional backwashing of filtered wastewater in facultative pond, concentrations of microorganisms in influent samples were initially lower (1–2 logs) than effluent samples in 2014 measurements (Table 3).

There was no significant decrease in numbers of microorganisms between the inlet and outlet of WSP3, based on samples collected during both sampling occasions in 2013 and 2014 (Table 3, Figure 2).

WSP4 consistently showed a difference of 0.5 log in faecal bacteria indicators concentrations between the influent and effluent sites in repeated samplings over two days (Table 3 and Supplementary Figure S1). Influent concentrations in WSP4 were similar to WSP1 for *E. coli* (1.7 ± 0.7 × 10^5^ CFU/100 mL), enterococci (2.6 ± 0.6 × 10^4^ CFU/100 mL) and higher for *Giardia* spp. (329.9 ± 7.6 cysts/L) and F+ RNA coliphages (3.4 ± 4.0 × 10^3^ PFU/100 mL) (Table 3). There was a statistically significant reduction in the number of *Giardia* cysts (1.3 log) and for F+ RNA coliphages (2.0 log) in effluent samples in this WSP. However, no reduction in the number of *Cryptosporidium* oocysts was observed (Figure 2). Adenovirus was analysed only once in WSP4 effluent with only 12.04 MPNIU per 10 L due to limited sampling possibilities at this remote location. Therefore removal rates were not determined for human adenovirus in this pond.

**Table 3:** Microbial concentrations and log removal values (LRV) in maturation ponds of four WSP systems tested over 2 years. Culture-based results are reported in CFU/PFU per 100 mL, qPCR counts are reported as genome copies per 100 mL.

Microorganism	Year	Method	WSP1, QLD (*n* = 8)			WSP2 *, QLD (*n* = 8)			WSP3, NT (*n* = 8)			WSP4, NT (*n* = 4)		
			Influent	Effluent	LRV	Influent	Effluent	LRV	Influent	Effluent	LRV	Influent	Effluent	LRV
*E. coli*	2013	culture	5.6 ± 0.6 × 10^4^	2.6 ± 0.7 × 10^1^	3.3	2.8 ± 0.07 × 10^2^	2.9 ± 0.4 × 10^2^	0.0	1.9 ± 0.01 × 10^4^	2.30 ± 0.01 × 10^4^	−0.1	NT	NT	-
		qPCR	6.6 ± 3 × 10^4^	2.2 ± 3.2 × 10^2^	2.5	7.7 ± 3.3 × 10^3^	3.64 ± 0.7 × 10^3^	0.3	7.6 ± 3.3 × 10^5^	4.1 ±1.12 × 10^5^	0.3			
	2014	culture	1.5 ± 0.2 × 10^5^	3.5 ± 0.8 × 10^2^	2.6	2 × 10^2^	ND	2.3	1.3 ± 0.01 × 10^5^	1.4 ± 0.1 × 10^5^	0.0	1.7 ± 0.7 × 10^5^	4.4 ± 0.5 × 10^4^	0.6
		qPCR	1.5 ± 0.1 × 10^6^	1.2 ± 6.6 × 10^3^	3.1	3.2 ± 0.8 × 10^4^	ND	-	1.7 ± 0.2 × 10^6^	2.07 ± 0.1 × 10^6^	−0.1	3.1 ± 0.0 × 10^6^	8.97 ± 0.06 × 10^5^	0.5
*Enterococcus* spp.	2013	culture	1.9 ± 0.05 × 10^4^	7.0 ± 4.2 × 10^2^	1.4	7.2 ± 0.2 × 10^2^	8.8 ± 1.6 × 10^2^	−0.1	4.7 × 10^3^	4.3 × 10^3^	0.0	NT	NT	-
		qPCR	2.7 ± 1.2 × 10^6^	1.9 ± 1.7 × 10^3^	3.2	1.6 ± 0.04 × 10^6^	3.4 ± 0.1 × 10^5^	0.7	3.2 ± 1.05 × 10^6^	2.16 ± 1.32 × 10^6^	0.2			-
	2014	culture	4.4± 0.07 × 10^4^	4.3 ± 0.6 × 10^4^	0.0	3.0 × 10^2^	ND	2.4	1.1 ± 0.6 × 10^3^	1.1 ± 0.3 × 10^3^	ND	2.8 ± 0.6 × 10^4^	9.1 ± 5.3 × 10^3^	0.5
		qPCR	1.5 × 10^7^	3 × 10^6^	0.7	4.4 ± 0.6 × 10^6^	9.1 ± 2.2 × 10^2^	3.7	1.9 ± 1.01 × 10^4^	1.5 ± 0.21 × 10^4^	0.1	2.5 ± 0.01 × 10^5^	4.9 ± 0.7 × 10^4^	0.7
*Salmonella* spp.	2013	culture	3.0 ± 0.4 ×10^3^	9.5 ± 7.8 × 10^1^	1.5	3.2 ± 0.2 × 10^2^	4.1 ± 0.8 × 10^2^	−0.1	NT	NT	-	NT	NT	-
		qPCR	ND	ND	-	ND	ND	-	ND	ND	-			
	2014	culture	5.5 ± 1.5 × 10^4^	3.4 ± 1.1 × 10^3^	1.2	ND	ND	0	NT	NT	-	NT	NT	-
		qPCR	ND	ND	-	ND	ND	-			-			
*Campylobacter* spp.	2013	qPCR	ND	ND	-	ND	ND	-	ND	ND	-	NT	NT	-
	2014	culture	7.0 × 10^1^	6.6 ± 5.7	1.0	ND	ND	-	9.6 ± 0.1 × 10^3^	8.0 ± 1.3 × 10^3^	0.1	NT	NT	-
		qPCR	ND	ND	-	ND	ND	-	ND	ND	-	ND	ND	
adenovirus	2014	qPCR	4.8 ± 1.5 × 10^3^	3.03 ± 1.3 × 10^3^	0.2	ND	ND	-	2.0 × 10^5^	2.4 × 10^5^	−0.1	NT	12.04 MPNIU	-
	2015	culture	23 MPNIU	6.6 MPNIU	1.2									
*Cryptosporidium* spp.	2014	microscopy	0.5 ± 0.7 (31%)	ND (22 %)	-	ND (16%)	ND (30%)	-	NT	NT	-	2 ± 1.7 (3%)	2.5 ± 1.4 (20%)	−0.3
	2015	microscopy	0.5 ± 0.7 (25%)	ND (24%)	-	NT	NT							
*Giardia* spp.	2014	microscopy	119 ± 130 (21%)	1 ± 1.4 (11%)	2.2	7.5 (18%)	ND (30%)	1.6	NT	NT	-	329.9 ± 7.6 (3%)	154.7 ± 30.7 (30%)	1.3
	2015	microscopy	76.5 ± 64.3 (26%)	ND (14%)	1.8									
F+ RNA coliphage	2013	culture	1.0 × 10^7^ ± 2.0	0	n/a	ND	ND	-	NT	NT	-	NT	NT	-
	2014	culture	<1.0 × 10^5^	2.0 × 10^1^	3.7	ND	ND	-	NT	NT	-	3.4 ± 4.0 × 10^3^	3.6 ± 4.9 × 10^1^	2.0
	2015	culture	9 × 10^3^	13	3.0									
Total coliforms	2013	culture	3.4 ± 0.2 × 10^4^	4.1 × 10^2^	1.9	8.8 ± 1.3 × 10^2^	5.9 ± 1.2 × 10^2^	0.2	NT	NT	-	NT	NT	-
	2014	culture	2.0 ± 0.1 × 10^5^	2.4 ± 0.2 × 10^3^	1.9	7.6 ± 0.2 × 10^3^	1.5 ± 1.0 × 10^2^	1.7	NT	NT	-	NT	NT	-

Values are presented by mean ± S.D. ND, Not detected; NT, Not tested; MPNIU—most probable number of infective units per 10 L, oocysts per 1 L with matrix recovery in percentage; *—maturation pond was affected by occasional backwashing during sampling period.

*S. enterica* and *C. jejuni* were not detected in 50 mL of influent and effluent samples by qPCR in any of the ponds (*Salmonella* spp. not tested in WSP4). PCR screening of 40 randomly selected colonies from mCCDA plates with the same marker for *C. jejuni* gave negative results indicating the lack of this pathogen in influent and effluent samples.

### 3.2. Further Analysis of Microbial Concentration of WSP1

**Figure 3:**
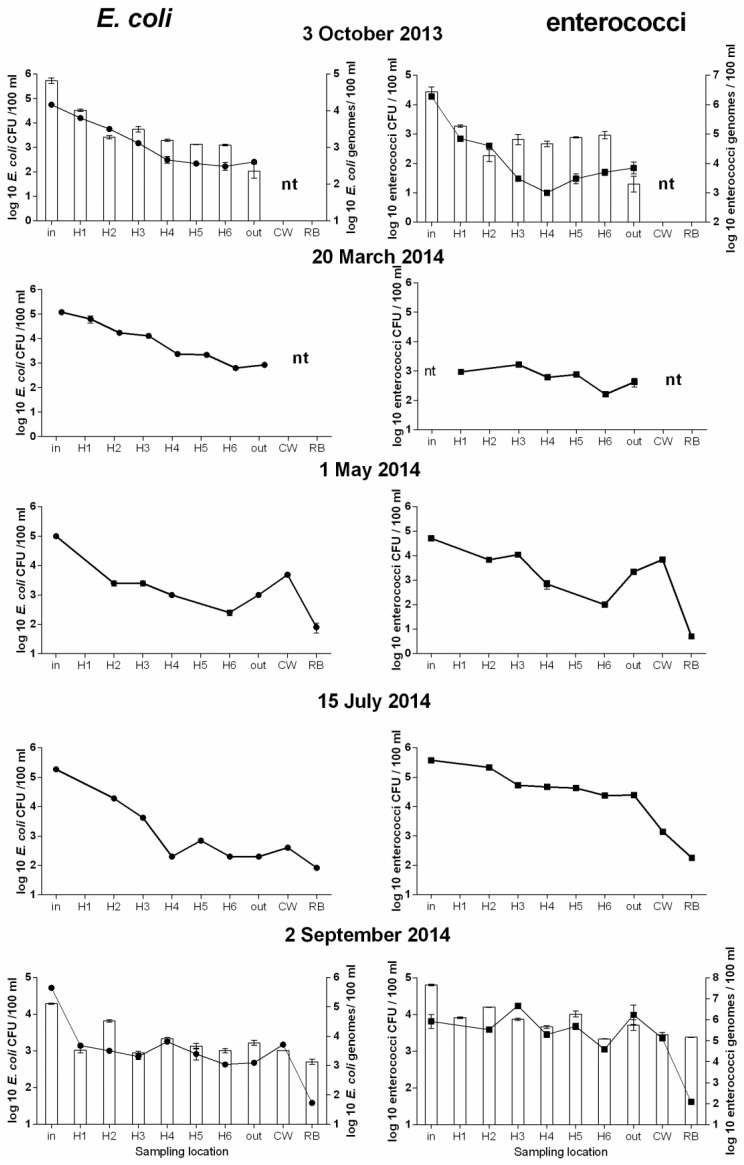
*E. coli* and enterococci concentrations analysed within maturation pond (influent, H1–H6, outlet) and effluents of constructed wetland (CW) and reed bed (RB) of WSP1. Samples were collected from 3 October 2013 to 2 September 2014. Dots—CFU/100 mL, bars qPCR genomes/100 mL. DNA was analysed only for October 2013 and September 2014, nt—samples were not taken at those locations, errors bars are for SD from biological duplicates.

Since the baffled WSP1 showed a greater reduction in the number of bacteria than the other unbaffled maturation ponds, an intensive sampling effort was undertaken between October 2013 and September 2014 from this WSP to measure the reduction in the number of these bacteria within the pond. From May 2014, additional samples from CW and RB sites were also included. The results of both culture-based analyses (performed on all samples) and qPCR (performed on samples collected at the beginning and at the end of the study) are presented in Figure 3. In general there was a gradual decrease in the number of both *E. coli* and enterococci toward the outlet and this pattern was consistent with both culture-based and qPCR methods (Figure 3). The number of *E. coli* and enterococci in the CW site showed an increase compared to the outlet and remained high in most samples indicating no removal within the CW. However, in July and September 2014, numbers of enterococci decreased in the CW (Figure 3). In contrast the RB site showed a high removal during the whole study period based on enumeration by culturing, whereas qPCR results indicated no removal at all. QPCR data for *E. coli* showed a significant high linear relationship (Pearson correlation coefficient 0.884, *p* value < 0.001, Spearman correlation coefficient 0.863) with corresponding CFU counts, whereas qPCR data for enterococci showed a lower but significant linear correlation with the corresponding CFU counts (Pearson correlation coefficient 0.651, *p* value = 0.016, Spearman correlation coefficient 0.540).

## 4. Discussion

### 4.1. Faecal Indicators and Pathogen Concentrations

To the best of our knowledge, this is the first study focused on assessing the microbial quality of remote and rural maturation ponds in Australia using both traditional and molecular methods. Based on microbiological analysis and retention time data in the four ponds studied, we suggest that baffled maturation pond WSP1 operates more effectively for pathogen removal (Figure 2), which is in line with previous studies [23,24]. This was demonstrated by a 3 log removal of *E. coli* counts in the baffled WSP1. Other WSPs analysed in this study lacked baffles which dramatically decreased the retention time and therefore we expected a lower removal rate in these WSPs as postulated before [1]. It has to be noted however that some factors such as sunlight penetration, temperature, pond length:width ratio, upstream treatment, degree of stratification, influent volume and strength as well as maintenance condition (*i.e.*, sludge accumulation) may also affect the performance of each pond.

*WSP2 maturation pond*. WSP2 had experienced multiple backwashing events from microfiltration plant to maturation pond on site during the year 2013 and 2014. Our data showed a 2.4 log bacterial removal for enterococci in 2014 by culture and 3.7 log by qPCR method. *E. coli* were below the detection limit in these effluent samples based on both methods (Table 3). One explanation might be that this reduction was likely due to dilution by backwashing events containing wastewaters which have been filtered and chlorinated rather than a true die-off of these bacteria. Operators on site might switch backwashing due to pressure in microfiltration plant and stop effluent coming from maturation pond. Furthermore, while concentrations of faecal indicators were approximately 2 × 10^2^ in the influent, no *Salmonella*, *Campylobacter* and adenovirus were detected. Despite that, *Giardia* spp. were present at 7.5 cysts per L with 18% recovery in the same samples which suggest a lack of correlation between the presence of faecal indicators and pathogenic bacteria but not protozoa. We also suggest that the presence and concentration of protozoa should be monitored for this pond if re-use of effluent is considered.

*WSP3 maturation pond.* WSP3, located at a remote aboriginal community in the Northern Territory, performed poorly during both sampling times, and it was since discovered that this pond was deeper than the expected 1.5 m. Stratification was observed in this pond (data not shown) as was high turbidity. These factors together with short (40 min) retention time would cause reduced light penetration and reduced mixing, which could explain the poor reduction.

*WSP4 maturation pond*. Among the non-baffled maturation ponds, only WSP4, which was the first of three maturation ponds (instead of one baffled pond), showed slight but consistent removal rates for faecal indicators. Interestingly this pond showed high removal rates of *Giardia* spp. and F+ RNA phage which was also observed in WSP1, suggesting that protozoa removal might be affected by cyst size which tend to settle if the retention time is long enough [25]. F+ RNA coliphage is known to be sensitive to light and affected by exogenous photo-oxidation [26] which may explain its high removal in all the studied ponds. Twelve infective adenoviral particles were detected in one liter of effluent of WSP4, however influent was not tested. As a result the removal efficiency of this pond for adenovirus could not be estimated. In our view and based on our data from WSP1, we believe that the F+ RNA phage is not as good a surrogate of viral removal compared to adenoviruses in maturation ponds due to the fact that it can be inactivated faster than adenovirus.

*Comparision of microbial removal between four ponds*. In most of the ponds, the removal of *E. coli* was much higher than enterococci despite similar initial concentrations at the inlet. Enterococci normally have a longer survival time than *E. coli* in surface waters [27,28] but have different mechanisms of inactivation in stabilization ponds [29,30]. Recently, it was found that enterococci could be inactivated faster than *E. coli* due to exogenous photo-oxidation [31]. Taking into account faster inactivation of enterococci in pond water, WSP1 maturation could be improved to favour exogenous mechanisms which are known to be responsible for inactivation of Gram-positive bacteria. Exogenous photo oxidation may be limited by the amount of available oxygen and mixing conditions. Adenovirus concentrations were very low (6–12 MPNIU per 10 L) in WSP1 effluent of which is very small compared to that reported for other wastewater treatment plants in Australia [32]. Culture-based methods counted only infective adenovirus, mainly genotype 2, which produces a cytopathic effect on A549 human cells lines. Thus, high numbers of adenovirus based on qPCR method could overestimate human health risk and culture based methods might underestimate that risk.

Different removal rates were observed for the two studied faecal indicators. Both bacteria showed mainly 2 log removal in their numbers through reed beds despite the fact that qPCR did not show any reduction in DNA markers. Difference in removal rates for the indicators used in this study were not comparable to the target pathogens in all WSP systems analysed in this study which suggests that direct assessment of actual pathogen removal rates in maturation ponds is needed for future validation of WSP performance.

In this study, F+ RNA coliphages were removed much faster than *E. coli* and despite their high concentrations in the inlet samples, their numbers in effluent samples were very low (if any at all). It is known that F+ RNA phages are inactivated by photo-oxidation and are sensitive to light [26]. The concentration of adenoviral DNA and infections adenovirus did not greatly change between influent and effluent samples suggesting that F+ RNA coliphage is not a good indicator of inactivation of adenovirus, as removal rates were significantly different.

Compared to bacterial removal rates, we found a high removal level of *Giardia* cysts in all maturation ponds, even in unbaffled WSP4 pond (1.3 logs) where there was a low reduction level for *E. coli*. It has been reported that these cysts settle in ponds due to sedimentation [25,33] although other factors such as sunlight and predation could also contribute to protozoa removal [34].

Low numbers of *Cryptosporidium* oocysts were detected in the inlet samples of all ponds but their recovery rates during laboratory processing were lower than that of *Giardia* (see Table 3). This could be partly due to the inefficiency of the method used to recover the oocysts or due to other factors such as interference of algae during the detection process or attachment of oocysts to suspended particles in samples making them difficult to bind to magnetic beads during the IMS process. Therefore, in our study we assessed protozoal removal efficiency of the pond based of the number of *Giardia* in the inlet and outlet samples. Protozoa removal rates revealed that there was no relationship with removal rates of faecal indicators in maturation pond studied, as found previously in Australia [35].

### 4.2. Comparison of qPCR Data and Culture Methods for Fecal Indicators

There was a high correlation between the number of gene copies and the number of cultures of *E. coli* and enterococci was based on two independent sampling events in various locations of the WSP1 (*p* value < 0.05). This however, was not the same for RB samples where the numbers of these bacteria in the culture method were lower than those showed by qPCR (Figure 3), suggesting that in RB neither one of the methods can substitute the other. Indicators could be inactivated but not removed in RB. Another explanation of differences between CFU numbers and qPCR could be that pathogens can enter viable but non-culturable (VBNC) state during wastewater treatment process. This is known result in up to a 5 log underestimation of viable pathogen numbers by culture methods [36]. The level of VBNC bacteria also depends on the pathogen species and the amount of stress such as oxygen or temperatures [37]. These data collectively suggest that detection of VBNC pathogens cannot to be ignored as this may affect the health risk assessment of surface waters or maturation pond where cells can retain their pathogenicity in the VBNC state [38,39]. Also inhibitory compounds which can be accumulated in reed beds could lead to underestimation of growth by cultivation [40]. Free DNA can exist in bound state with cations which protect DNA from nucleases in the environment [41] suggesting the use of both culture-based method and the qPCR should be used in such studies [42].

### 4.3. Salmonella *spp.* and Campylobacter *spp.* Enumeration

Variation in numbers of *Salmonella* spp. was observed due to difficulties with currently available enumeration methods for this pathogen from wastewater. This variation could be explained partially due to the growth and interference of other pond bacteria with visual detection of characteristic *Salmonella* spp. colonies on XLD medium. Therefore the use of selective media for estimation of low number of *Salmonella* spp. in pond samples may not always provide an ideal approach for their enumeration. This has also been reported by others [20,43]. To overcome this problem we employed the modified mini MPN method with enrichment. While this improved the detection of *Salmonella*, we found it too laborious to be used in an intensive study. *Salmonella enterica* was not detected by qPCR using either the *gyr*B or *tt*RSBCA genetic markers, despite the relatively high concentrations detected by the MPN method which included enrichment step. Applying an enrichment step prior qPCR could increase the detection limit of *Salmonella* a 1000 fold [44], but it could also bring extra variability in the interpretation of qPCR results due to the inability to quantify the initial concentration. In our study, the low signal obtained from qPCR could be explained by the initial low numbers of *Salmonella enterica* in pond samples. Levels from 0 to 1000 gene copies per mL of *inv*A marker have been reported for influent in wastewaters treatment plants with significant removal in effluent to below detection limit [36,45]. In some cases, the high levels of *inv*A marker in wastewaters might be an overestimation due to its cross-reactivity with *E. coli*. In the present study, we did not detect *S. enterica* in WSP1 samples using our probes or *gyr*B primers [20] which eliminates cross-reactivity with *E. coli*.

Despite the high numbers of thermotolerant *Campylobacter* spp. colonies detected on mCCDA agar, qPCR did not detect any *C. jejuni* markers in influent and effluent samples in any of the studied ponds which is in agreement with other reports [46]. One possible reason for this could be that mCCDA medium may lack specificity to detect *C. jejuni* in environmental samples. It is known that other bacterial species such as *E. coli*, *Proteus* spp., *Acinetobacter* spp. [47] as well as *Helicobacter* spp., *Arcobacter* spp., *Sutterella wadswortheness* [48] and *Bordetella pertussis* can also grow on mCCDA agar. Under microaerophilic conditions some of these bacteria may produce colonies of similar morphology to other pathogens such as *C. coli* and *C. lari*. It has to be noted however, that there is a limitation with the current qPCR protocol for the detection of the VS1 marker. This marker has been used extensively in several environmental studies and has shown a low detection limit of 200 cells per mL or even 10 times lower sensitivity as reported by others [49,50].

It is expected that a well-designed and maintained system can give high pathogen removal values, however this might be subjected to seasonal variations. In this study although we found that maturation pond with baffles performs much better in removal of indicator bacteria than non-baffled ponds, we believe that more sampling data will be needed to characterize the performance of maturation ponds in remote areas over different seasons. This is particularly important when the aim is to understand the relationship between faecal indicators and pathogens.

## 5. Conclusions 

Among four maturation ponds analysed in rural and remote Australia, only the baffled pond had significant log removal of fecal bacteria, viruses and protozoa which could be mainly due to the longest retention time of this pond. *E. coli* and enterococci cannot be used as surrogates for pathogenic bacteria or protozoa, and F+ RNA phage cannot be used as a surrogate for adenovirus to study pond disinfection. Enumeration by either qPCR or culturing gave similar log removal rates in case of *E. coli* with less confidence for enterococci in maturation pond. There was a significant linear correlation between qPCR and culturing for *E. coli* and enterococci. Standard selective medium employed in these studies for quantification of *Campylobacter* spp. and *Salmonella* spp. led to mis-interpretation of their actual concentrations in maturation pond analysed. In the baffled pond, most of the pathogens were removed within the first few baffles as there was no (if any at all) removal of bacteria in samples collected from the middle of the pond onward. Viable bacteria were removed by reed beds but the constructed wetlands were not efficient most of the time. In the reed beds, there was no correlation between the number of bacteria estimated with culture methods and the qPCR.

## Supplementary Files

Supplementary File 1
