# Supplementary material for: Removal of Fecal Indicators, Pathogenic Bacteria, Adenovirus, Cryptosporidium and Giardia (oo)cysts in Waste Stabilization Ponds in Northern and Eastern Australia
Source: Int J Environ Res Public Health. 2016 Jan 2;13(1):96. doi: 10.3390/ijerph13010096 (PMC4730487; doi:10.3390/ijerph13010096)
Supplement: Supplementary File 1 [file ijerph-13-00096-s001.pdf]

Supplementary Materials: Removal of Fecal Indicators, Pathogenic Bacteria, Adenovirus, *Cryptosporidium* and *Giardia* (oo)cysts in Waste Stabilization Ponds in Northern and Eastern Australia

Maxim Sheludchenko, Anna Padovan, Mohammad Katouli and Helen Stratton

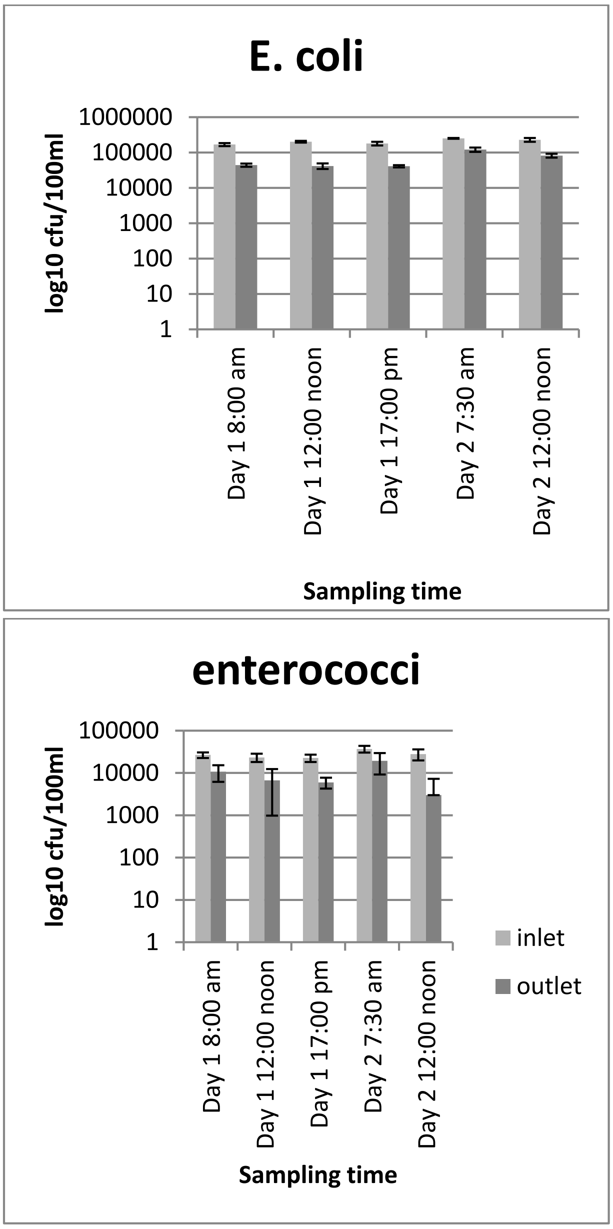

Figure S1. Concentrations of *E. coli* and enterococci at the inlet and outlet of WSP4 over a day and a half. Error bars are standard deviation taken from triplicate samples.

© 2015 by the authors; licensee MDPI, Basel, Switzerland. This article is an open access article distributed under the terms and conditions of the Creative Commons by Attribution (CC-BY) license (http://creativecommons.org/licenses/by/4.0/).
